# Brain morphometric features predict medication response in youth with bipolar disorder: a prospective randomized clinical trial

**DOI:** 10.1017/S0033291722000757

**Published:** 2023-07

**Authors:** Du Lei, Kun Qin, Wenbin Li, Walter H. L. Pinaya, Maxwell J. Tallman, L. Rodrigo Patino, Jeffrey R. Strawn, David Fleck, Christina C. Klein, Su Lui, Qiyong Gong, Caleb M. Adler, Andrea Mechelli, John A. Sweeney, Melissa P. DelBello

**Affiliations:** 1 Department of Psychiatry and Behavioral Neuroscience, University of Cincinnati College of Medicine, Cincinnati 45219, OH, USA; 2 Huaxi MR Research Center (HMRRC), Department of Radiology, West China Hospital of Sichuan University, Chengdu 610041, PR China; 3 Department of Biomedical Engineering, School of Biomedical Engineering & Imaging Sciences, King's College London, Westminster Bridge Road, London, UK; 4 Department of Psychosis Studies, Institute of Psychiatry, Psychology & Neuroscience, King's College London, De Crespigny Park, London, UK

**Keywords:** Deep learning; bipolar disorder; MRI; cortical thickness; treatment; Young Mania Rating Scale

## Abstract

**Background:**

Identification of treatment-specific predictors of drug therapies for bipolar disorder (BD) is important because only about half of individuals respond to any specific medication. However, medication response in pediatric BD is variable and not well predicted by clinical characteristics.

**Methods:**

A total of 121 youth with early course BD (acute manic/mixed episode) were prospectively recruited and randomized to 6 weeks of double-blind treatment with quetiapine (*n* = 71) or lithium (*n* = 50). Participants completed structural magnetic resonance imaging (MRI) at baseline before treatment and 1 week after treatment initiation, and brain morphometric features were extracted for each individual based on MRI scans. Positive antimanic treatment response at week 6 was defined as an over 50% reduction of Young Mania Rating Scale scores from baseline. Two-stage deep learning prediction model was established to distinguish responders and non-responders based on different feature sets.

**Results:**

Pre-treatment morphometry and morphometric changes occurring during the first week can both independently predict treatment outcome of quetiapine and lithium with balanced accuracy over 75% (all *p* < 0.05). Combining brain morphometry at baseline and week 1 allows prediction with the highest balanced accuracy (quetiapine: 83.2% and lithium: 83.5%). Predictions in the quetiapine and lithium group were found to be driven by different morphometric patterns.

**Conclusions:**

These findings demonstrate that pre-treatment morphometric measures and acute brain morphometric changes can serve as medication response predictors in pediatric BD. Brain morphometric features may provide promising biomarkers for developing biologically-informed treatment outcome prediction and patient stratification tools for BD treatment development.

## Introduction

Bipolar disorder (BD) frequently emerges during adolescence (Lewinsohn, Klein, & Seeley, 2000; Lewinsohn, Seeley, Buckley, & Klein, 2002; Perlis et al., 2004). Identification of treatment-specific predictors of drug therapies is important because only about half of individuals with BD respond to any specific medication (Hirschfeld et al., 2004; Keck et al., 2003; Keck, Welge, Strakowski, Arnold, & McElroy, 2000). Reliable predictive models could guide personalized therapeutics and implementation of supplemental drug and psychotherapeutic interventions to decrease the high levels of morbidity and mortality associated with the disorder.

Interest in identifying neuroimaging biomarkers of treatment outcome in BD has received increased attention over the past two decades (Ketter & Wang, 2002; Lim et al., 2013). For example, Passarotti, Sweeney, and Pavuluri (2011) used task-based functional magnetic resonance imaging (MRI) to predict the clinical effects of antipsychotic treatment in pediatric BD. Moore et al. (2009) found total brain gray matter volume was increased in treatment-responsive adults with BD after 4 weeks of lithium treatment. Fleck et al. (2017) used magnetic resonance spectroscopy to predict medication response in patients with BD, and Zhang et al. (2018) showed that pre-treatment cortical thickness measures predicted response to an antipsychotic drug in youth with BD. Among these measures, brain morphometric measures have been most widely used to predict and track the effects of drug therapies for psychiatric disorders (Hazlett et al., 2017; Vieira et al., 2020).

Quetiapine and lithium are effective and widely used treatments for BD. Different neuroprotection effects between quetiapine and lithium have been reported in previous studies. Specifically, lithium was found to be more effective than quetiapine in slowing the progression of white matter abnormalities (Berk et al., 2017). Dandash et al. found that lithium exhibited higher efficiency in reversing the hyperconnectivity of striatal areas than quetiapine (Dandash et al., 2018). For brain gray matter morphometry, our previous work suggested that discrete patterns of baseline cortical thickness in temporal and parietal regions differentially predicted treatment response of quetiapine but not lithium (Zhang et al., 2018). Given the different neural pathways impacted by quetiapine and lithium, comparing the discriminative regions in these two predictive models may provide insights into the neuropharmacological mechanisms of clinical efficacy of the two drugs. While there has been a study using clinical features as predictors to build prediction models for these two drugs separately (Kim et al., 2019), models based on MRI features have not yet been established.

Most studies of neuroimaging predictors of treatment outcome have examined individuals with long-term BD who had received extensive treatment in the past. Therefore, some findings in prior work may have been impacted by the effects of chronicity and previous or current medications. Studying recent onset cases with limited treatment history may better illuminate medication effects on illness processes and predictive brain features, as well as provide more clinically useful information. Further, studying early course individuals with BD may provide more clinically useful information because an extensive clinical history is not available to guide treatment decisions (Brooks & Vizueta, 2014). Third, focusing on patients experiencing an acute mixed/manic episode can facilitate assessment of MRI features in relation to treatment outcome. Fourth, preclinical studies have revealed changes in brain systems induced by short-term administration of lithium and quetiapine. For example, 7 days of lithium therapy resulted in changes in forebrain membrane properties in a rat model of mania (Vošahlíková, Roubalová, Brejchová, Alda, & Svoboda, 2021), and 1-week treatment of quetiapine was found to enhance the level of hippocampal neurogenesis (Luo, Xu, & Li, 2005). In addition, evidence from clinical trials based on neuroimaging techniques also support brain changes following short-term quetiapine or lithium therapy. Brain D2 dopamine receptor occupancy can be observed following acute quetiapine treatment (Nord et al., 2011), and significant decreases in myoinositol levels were identified in the right frontal cortex after 5–7 days of lithium administration (Moore et al., 1999). Moreover, our previous study using brain structural MRI found youth with BD exhibited changes in brain structural network following 1-week quetiapine or lithium treatment (Lei et al., 2021). Although these findings suggest that brain changes can occur after acute drug treatment, few studies investigated whether such acute brain changes can serve as predictors of treatment outcome.

Previous studies have suggested that deep learning, a type of machine learning capable of capturing high orders of complexity and abstraction (Kim, Calhoun, Shim, & Lee, 2016; LeCun, Bengio, & Hinton, 2015; Lei et al., 2020), may yield higher classifier accuracy than the current widely adopted traditional machine learning models (Pinaya et al., 2016; Vieira, Pinaya, & Mechelli, 2017). It has been reported that brain structure is undergoing non-linear trajectory of brain structural maturational changes in typically developing youth (Giedd et al., 1999; Tamnes et al., 2017). Previous studies have also found non-linear characteristics in brain alterations associated with BD and increased brain complexity in mania (Bahrami, Seyedsadjadi, Babadi, & Noroozian, 2005; Fernández, Al-Timemy, Ferre, Rubio, & Escudero, 2018). Considering additional complicated neuroprogression and medication effects, non-linear models may be better positioned to address the brain complexity involved in a randomized clinical trial of pediatric BD compared with linear methods (e.g. principal component analysis, sparse learning) (Hazlett et al., 2017). Deep neural network can precisely recognize the most differentiable features related to medication response from the complex longitudinal structural patterns in a non-linear way, and facilitate the extraction of optimal low-dimensional representations for clinicians unequipped with expert feature engineering knowledge. We therefore utilized a two-stage prediction pipeline that includes a deep neural network component for non-linear dimensionality followed by an additional support vector machine (SVM) classifier (Hazlett et al., 2017).

With these considerations in mind, we recruited a cohort of young participants with BD who were early in their illness course and randomized to receive quetiapine or lithium trial. Our aims were to determine the utility of pre-treatment morphometric data and change in brain structure after 1 week of treatment for the prediction of treatment response at the end of the 6 weeks trial. We hypothesized that (i) baseline image data could significantly predict medication response in bipolar youth at the individual level; and (ii) change in morphometric features from baseline to 1 week after treatment initiation would predict treatment response, and the longitudinal joint model combining baseline data and week 1 structural changes data would predict medication response with highest accuracy. Finally, considering that the drug mechanisms of lithium and quetiapine are different (Ketter, Miller, Dell'Osso, & Wang, 2016), we hypothesized that (iii) pre-treatment morphometric features and their change may be differentially related to outcome prediction in the two treatment groups.

## Methods

### Participants

This study was approved by the University of Cincinnati and the Cincinnati Children's Hospital Medical Center Institutional Review Boards. Youth with bipolar I disorder were recruited from the Cincinnati Children's Hospital Medical Center, the University of Cincinnati, and the local community. Diagnosis of bipolar I disorder was confirmed using the Washington University in St. Louis Kiddie Schedule of Affective Disorders and Schizophrenia administered by raters with demonstrated inter-rater reliability (*κ* > 0.9) (Geller et al., 2001). Young Mania Rating Scale (YMRS) was used to assess mania symptoms (Young, Biggs, Ziegler, & Meyer, 1978). Written informed assent and consent about the study procedures and purpose were provided by all participants and their legal guardians (registered in https://clinicaltrials.gov/; registration number: NCT00893581).

To be included, participants were required to be: (1) within the age range of 10–18 years old; (2) experiencing a manic or mixed episode; (3) having a baseline YMRS score ⩾20; (4) within 2 years from onset of BD; (5) having no prior psychiatric hospitalizations for mania; (6) having no treatment history with therapeutic doses of antipsychotics or mood stabilizers for over 3 months, and no psychotropic medication during the week prior to baseline scans. Participants were excluded if they had (1) a contraindication to MRI scanning; (2) an IQ <70; (3) a positive pregnancy test; (4) a history of a major systemic or neurological illness, or an episode of loss of consciousness >10 min; (5) any lifetime DSM-IV-TR substance use disorder (nicotine dependence was permitted); or (6) a lifetime DSM-IV-TR diagnosis of a pervasive developmental disorder or post-traumatic stress disorder.

### Treatment procedures

Following initial clinical evaluation and MRI scanning, participants were randomized by an investigational pharmacist (C.C.K.) to double-blind treatment with lithium or quetiapine and evaluated clinically weekly for 6 weeks. The randomization schedule was stratified by presence/absence of attention deficit and hyperactivity disorder, presence/absence of psychosis, and the mood state (mixed *v*. manic episode). Quetiapine was initiated at 100 mg qhs and lithium carbonate was initiated at 30 mg/kg (maximum starting dose of 600 mg twice daily). Patients were also given placebo capsules for the medication to which they were not assigned. Quetiapine was titrated to a target dose of 400–600 mg/day based on tolerability and response. Lithium was titrated to a serum level of 1.0–1.2 mEq/L. Treatment was administered in a double-dummy, double-blind manner, with an unblinded study psychiatrist monitoring trough lithium levels and making dose adjustments independent from treating psychiatrist and clinical raters. Blinded clinical tolerability dose adjustment recommendations took precedent over un-blinded double dummy dose adjustment recommendations. There were no significant changes of the treatment methods or outcomes after trial commencement. We used the YMRS changes to assess antimanic treatment response at week 6, and treatment responder was defined as exhibiting ⩾50% reduction in YMRS scores from baseline (Patino et al., 2021; Wegbreit et al., 2011).

### MRI acquisition

MRI scanning was performed on a 4-T Varian Unity INOVA scanner using a 12-channel head coil at baseline. All participants were instructed to be scanned at baseline prior to treatment and 1 week after treatment initiation. Earplugs and headphones were provided to reduce background noise, and foam padding around the head minimized head motion. Following a three-plane gradient echo scan for alignment and localization, a shim procedure was performed to generate a homogeneous magnetic field. High-resolution T1-weighted three-dimensional images were acquired with a Modified Driven Equilibrium Fourier Transform (MDEFT) protocol, optimized for the 4T Varian scanner [Tau (magnetization preparation time) = 1.1 s, TR = 13 ms, TE = 5.3 ms, field of view = 192 × 256 × 256 mm, matrix = 192 × 256 × 256, flip angle = 20 degrees, slice thickness = 1 mm]. Acquired T1-weighted images were inspected by two experienced neuroradiologists who made decisions about excessive motion artifact for scan exclusion. No observable scanning artifacts or gross brain abnormalities were identified in any participant included in the following analyses.

### Image processing and brain morphometric feature sets

All structural MRI scans were processed on the same workstation using the FreeSurfer image analysis suite v6.0.0 (http://surfer.nmr.mgh.harvard.edu/) to obtain unbiased estimates of morphometric measures, including surface area, cortical thickness, and subcortical volumes (for detailed calculation and extraction of these morphometric measures, see online Supplementary Materials). Each participant was thus represented by a 150-dimensional feature vector consisting of morphometric features across the whole brain. Specifically, we included the surface area and cortical thickness of 68 cortical regions labeled in the Desikan/Killiany Atlas (Desikan et al., 2006), as well as the volume of bilateral hippocampus and 12 subcortical regions (i.e. bilateral thalamus, amygdala, caudate, putamen, pallidum, and accumbens). To compare the prediction value of baseline brain structure and acute structural alterations induced by medication, the model performance was investigated for baseline morphometric measures and morphometric changes from baseline to week 1 independently. To thoroughly take advantage of longitudinal brain morphometry related to antimanic treatment for better model performance, we further combined baseline and week 1 morphometric features into a concatenated 300-dimensional vector as our longitudinally joint model. Therefore, under the unified prediction framework, there are finally three classification models based on different feature sets (i.e. baseline model, 1-week change model, and longitudinally joint model) to be tested in two medication groups.

### Two-stage prediction model based on structural MRI

We implemented a two-stage prediction pipeline to differentiate medication responders from non-responders as described in previous prospective studies (Hazlett et al., 2017; Zhu et al., 2021; Yang et al., 2021). A feedforward multi-layer neural network was adopted as the initial stage for dimensionality reduction (Hinton & Salakhutdinov, 2006), and SVM was included as the second stage to individually discriminate responders from non-responders (Cortes & Vapnik, 1995). The training procedures of neural network for dimensionality reduction mainly contain binary processing of raw features, unsupervised pre-training of stacked autoencoders, and supervised training of fine-tuned neural network. Once the training was finished, the representations stored in the layer before output layer were extracted as the optimal features fed into SVM. Detailed information about the rationale, architecture, and training process of the two-stage prediction model is shown in online Supplementary Materials.

The whole two-stage prediction pipeline was trained and evaluated using 10-fold stratified cross-validation. For each iteration of the cross-validation, we used one part of the dataset to evaluate our model (i.e. testing set) and the remaining nine parts for training (i.e. training set). To avoid information leakage, we performed the non-linear dimensionality reduction (i.e. training of neural network) only based on the training set, and the testing set was only used to evaluate performance and never used for tuning or training our models. The model performance was determined by the balanced accuracy, sensitivity, specificity, and area under receiver operating characteristic curve (AUC). We independently reported the model performance in quetiapine and lithium medication group. All the machine learning analyses were programmed using Python language, where the neural network was implemented in the Pytorch library (Paszke et al., 2019), and the SVM was implemented in the Scikit-Learn library (Pedregosa et al., 2011). All the codes are available at https://github.com/QKmeans0902/Two_stage_prediction_pipeline.

### Model transferability between different medication groups

In this analysis, we examined the model transferability between two medication groups. Specifically, we used dataset from one whole treatment group (quetiapine or lithium) for drug-specific model training and optimization, and the other group to evaluate the predictive utility of the model for treatment outcome to the other drug therapy. To maintain consistency with preceding analyses, model transferability test was also implemented in the identical two-stage prediction pipeline.

### Features with greatest contributions to predicting medication outcomes

Given morphometric features with the highest predictive power may differ in different medication groups which can provide useful neurobiological implications, we sought to identify these potentially discriminating patterns. Detailed calculation of feature contribution is presented in online Supplementary Materials. Top 10 features with greatest contributions are reported for different models and medication groups.

### Statistical analysis

Group comparisons of demographic and clinical characteristics were performed using SPSS software [version 23 (IBM SPSS Statistics)]. The significance level of prediction model was evaluated using permutation test (Golland & Fischl, 2003). Specifically, we randomly permuted the labels of training set prior to training. The model training and evaluation under cross-validation were then performed to get the estimated accuracy based on the permuted dummy training set. The permutation was repeated 1000 times, and a distribution of the accuracy for randomly permuted data was obtained. The significance level was thus defined as the probability of observing an accuracy from the distribution based on permuted data no less than the real accuracy. Differences in age and IQ were assessed by two-sample *t* test, while differences in sex and number of responders were assessed using χ^2^ test. The *p* value of differences in parental socioeconomic status was calculated by Mann–Whitney *U* test given the non-normality, and the statistical test for differences in longitudinal clinical scale scores was two-way repeated measures ANOVA.

## Results

### Demographic and clinical characteristics

This prospective randomized clinical trial recruited 149 youth with BD. Data from nine participants were excluded due to failure to finish baseline structural MRI scan or excessive head motion. Eleven participants in the lithium treatment group (*n* = 61) and eight participants in the quetiapine treatment group (*n* = 79) were lost to follow-up. Ultimately, 71 participants treated with quetiapine and 50 participants treated with lithium who had completed all follow-up structural MRI examinations and clinical assessments were finally included in the analysis.

The demographic and clinical characteristics are listed in Table 1 and online Supplementary Table S1. No evidence of significant differences in age, sex, IQ, parental socioeconomic status, or number of responders was found between the quetiapine and lithium groups (all *p* > 0.05). No significant time × treatment group interaction effects in YMRS scores were observed. For the group effect, we found no significant between-group differences in YMRS scores at any follow-up timepoint (*p* > 0.05). For the time effect, the YMRS scores were significantly decreased from baseline to week 6 in both quetiapine and lithium groups (*p* < 0.001).

**Table 1.** Demographic and clinical characteristics of youth with bipolar disorder

Characteristic	Quetiapine (*n* = 71)	Lithium (*n* = 50)	*p* value
Age (years)^1^	14.84 ± 1.62 (10.2–17.7)	15.44 ± 1.72 (10.5–17.8)	0.053
Sex (female/male)	46/25 (65.0%/35.0%)	35/25 (70.0%/30.0%)	0.548
IQ	102.57 ± 12.14	103.97 ± 11.37	0.599
PSES	2.98 ± 1.11	2.97 ± 1.00	0.969^2^
BMI	23.85 ± 5.53	25.42 ± 7.19	0.198
Duration of current episode (weeks)^3^	19.39 ± 27.95	15.49 ± 23.56	0.430
No. of lifetime episodes^2^	1.31 ± 1.90	0.74 ± 1.03	0.066
Treatment response, *N* (%)			0.531
Responder	52 (73.2%)	34 (68.0%)	
Non-responder	19 (26.8%)	16 (32.0%)	
Mood state, *N* (%)			0.660
Mania	44 (62.0%)	29 (58.0%)	
Mixed	27 (38.0%)	21 (42.0%)	
YMRS			0.580^4^
Baseline	26.42 ± 5.06	26.34 ± 5.13	
Week 1	15.44 ± 6.36	16.56 ± 8.18	
Week 6	9.64 ± 6.84	10.83 ± 6.86	
CDRS-R			0.981^4^
Baseline	37.73 ± 9.05	38.50 ± 8.98	
Week 1	31.33 ± 6.97	31.79 ± 9.19	
Week 6	27.06 ± 8.33	27.88 ± 6.95	
CGI-S			0.983^4^
Baseline	4.84 ± 0.59	4.94 ± 0.55	
Week 1	3.82 ± 0.75	3.94 ± 0.92	
Week 6	2.86 ± 1.26	3.00 ± 1.23	

IQ, intelligence quotient; PSES, parental socioeconomic status; BMI, body mass index; YMRS, Young Mania Rating Scale; CDRS-R, Children's Depression Rating Scale - Revised; CGI-S, Clinical Global Impressions-Severity.

*Note:* Data are presented as mean ± standard unless otherwise indicated. The *p* values were calculated by two-sample *t* test or χ^2^ test unless otherwise indicated.

1 Age are presented as mean ± standard with age range in parenthesis.

2 Data were available in 111 of 121 participants.

3 Data were available in 116 of 121 participants.

4 The *p* values refer to significance level of time × treatment group interaction effects using two-way repeated measures ANOVA.

### Prediction performance of medication response

**Fig. 1.**
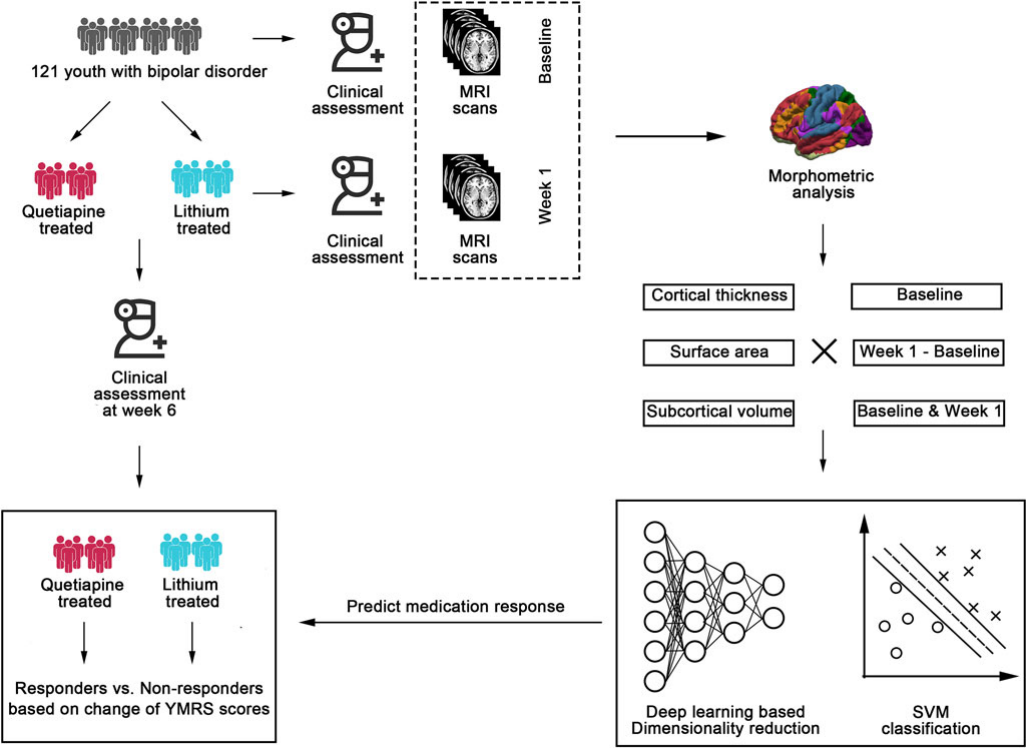
The pipeline of treatment response prediction. A total of 121 youth with BD were included and randomly assigned to quetiapine and lithium treatment group. Structural MRI examination was performed prior to and at week 1 of the treatment. Clinical assessments were implemented at baseline, week 1, and week 6, respectively. To develop a medication response prediction model using structural MRI data, we extracted the morphometric measures including cortical thickness, surface area, and subcortical volume. Responders were determined as a reduction of YMRS scores >50% at week 6. Baseline, change during the first week (baseline – week 1), and longitudinally combined morphometric features (baseline + week 1) were separately investigated for both medication groups. The two-stage prediction model including non-linear dimensionality reduction and support vector machine classifier was applied consistently. SVM, support vector machine; YMRS, Young Manic Rating Scale.

The prediction pipeline in the current study was shown in Fig. 1. Baseline structural MRI predicted treatment response at week 6 with balanced accuracy of 79.6% (AUC: 0.89, *p* < 0.001) for the quetiapine treatment group. For the lithium treatment group, baseline morphometric measures achieved balanced accuracy of 75.8% (AUC: 0.85, *p* = 0.003). When using 1-week brain morphometric changes as predictors, balanced accuracies in the quetiapine and lithium treatment group were 76.5% (AUC: 0.81, *p* < 0.001) and 78.9% (AUC: 0.85, *p* < 0.001), respectively. Combining brain morphometric features at baseline and week 1 allowed for the highest prediction performance. For the quetiapine treatment group, the balanced accuracy was 83.2% (AUC: 0.93, *p* < 0.001). For the lithium treatment group, the balanced accuracy was 83.5% (AUC: 0.89, *p* < 0.001). All the evaluation metrics for model performance were listed in Table 2.

**Table 2.** Model classification and transferability performance between quetiapine and lithium treatment groups

Model performance	BAC, %	SEN, %	SPE, %	AUC
Baseline model				
Quetiapine	79.6	75.0	84.2	0.89
Lithium	75.8	76.5	75.0	0.85
Quetiapine model predicts lithium outcome	54.4	58.8	50.0	0.51
Lithium model predicts quetiapine outcome	55.7	69.2	42.1	0.49
One-week change model				
Quetiapine	76.5	63.5	89.5	0.81
Lithium	78.9	73.5	81.3	0.85
Quetiapine model predicts lithium outcome	43.8	50.0	37.5	0.47
Lithium model predicts quetiapine outcome	47.7	48.1	47.4	0.50
Longitudinally joint model				
Quetiapine	83.2	76.9	89.5	0.93
Lithium	83.5	79.4	87.5	0.89
Quetiapine model predicts lithium outcome	51.5	52.9	50.0	0.58
Lithium model predicts quetiapine outcome	48.9	55.8	42.1	0.53

BAC, balanced accuracy; SEN, sensitivity; SPE, specificity; AUC, area under receiver operating characteristic curve.

### Model transferability analyses between drug treatments

All models failed to reach comparable performance in the transferability test when prediction models developed for one drug were applied to the other drug treated group (all *p* > 0.05; Table 2). Baseline morphometric measures showed slightly above chance level performance (i.e. 50%) using the quetiapine model to predict lithium treatment outcome (balanced accuracy, 54.4%) and *vice versa* (balanced accuracy, 55.7%). Using week 1 structural change data, both models showed poor transferability below chance level (balanced accuracy of 43.8% for quetiapine model predicting lithium outcome and balanced accuracy of 47.7% for lithium predicting quetiapine outcome). When considering both baseline and week 1 morphometric data together, both models transferred around chance level (quetiapine model predicting lithium outcome: balanced accuracy, 51.5%; lithium model predicting quetiapine outcome: balanced accuracy, 48.9%).

### Features with greatest contributions to treatment response prediction

**Fig. 2.**
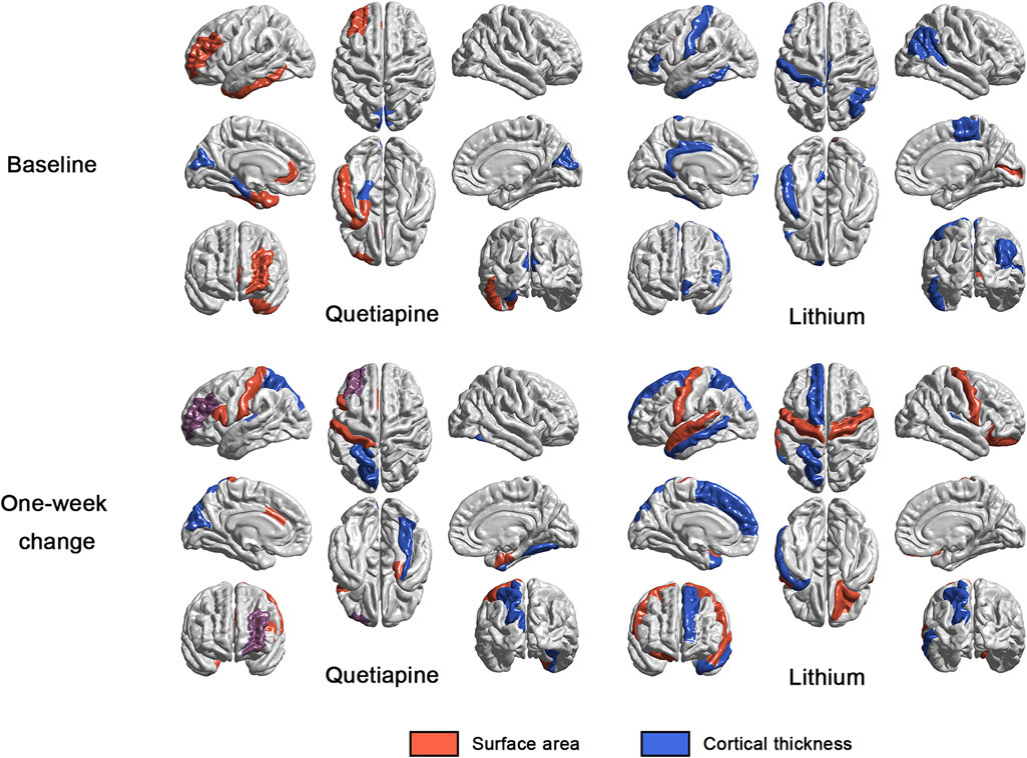
Cortical regions of surface area and cortical thickness measures among top 10 morphometric features contributing to the non-linear dimensionality reduction. For each model, results were independently showed in both quetiapine and lithium group. Surface area measures are shown in red, and cortical thickness measures are shown in blue. If both cortical thickness and surface area of a single region exhibit top 10 contribution, this region will be shown in a hybrid purple color combining blue and red.

Within the models considering the predictive utility of pre-treatment morphometric features, top predictive features in the lithium group mostly included cortical thickness measures, while in the quetiapine group, cortical thickness, cortical surface area, and subcortical volume measures were leading predictors. When using acute structural changes from baseline to week 1 for prediction, top features in the lithium group included cortical surface area and hippocampal volume, while in the quetiapine group, the leading predictors were cortical thickness measures (Table 3 and Fig. 2).

**Table 3.** Top 10 morphometric features showing greatest contribution to baseline and 1-week change model

Rank	Quetiapine		Lithium	
	Measure	Region	Measure	Region
*Baseline model*				
1	CT	Cuneus L	CT	Inferior temporal L
2	Vol	Accumbens L	CT	Paracentral R
3	SA	Entorhinal L	CT	Isthmus L
4	SA	Temporal pole L	CT	Frontal pole L
5	Vol	Hippocampus L	CT	Inferior parietal R
6	CT	Parahippocampal L	CT	Postcentral L
7	SA	Inferior temporal L	SA	Pericalcarine R
8	CT	Cuneus R	CT	Posterior cingulate L
9	SA	Rostral anterior cingulate L	CT	Pars triangularis L
10	SA	Rostral middle frontal L	CT	Banks of superior temporal sulcus R
*One-week change model*				
1	CT	Cuneus L	SA	Precentral R
2	CT	Transverse temporal L	CT	Temporal pole L
3	SA	Pars opercularis L	CT	Superior frontal L
4	CT	Rostral middle frontal L	CT	Superior parietal L
5	SA	Postcentral L	SA	Precentral L
6	SA	Rostral middle frontal L	Vol	Hippocampus L
7	CT	Fusiform R	SA	Lateral orbitofrontal R
8	SA	Entorhinal R	SA	Superior temporal L
9	SA	Caudal anterior cingulate L	CT	Middle temporal L
10	CT	Superior parietal L	CT	Transverse temporal R

L, left; R, right; CT, cortical thickness; SA, surface area; Vol, volume.

## Discussion

In the present study, we tested the ability of brain morphometric features to predict medication treatment response in youth with BD. We know of only two previous studies that applied pre-treatment MRI data to predict treatment outcome in BD: Wade et al. (2016) trained an SVM with subcortical volume and cortical thickness features to predict response to electroconvulsive therapy in depressed adults, eight of whom had BD. They reported an accuracy of 89%. Fleck et al. (2017) trained a machine learning model with pre-treatment fMRI and magnetic resonance spectroscopy data to predict response to lithium at week 8 in 20 adults with BD, classifying responders *v.* non-responders with 80% accuracy. Our study is considerably larger, which focused on youth with limited clinical and treatment history and considered acute neuroanatomic changes after 1 week of treatment as well as baseline predictors.

Consistent with our first hypothesis, we found that baseline morphometric measures predicted medication response at the individual level with significant accuracy, 79.6% for the quetiapine group and 75.8% for the lithium group. Our findings indicate that pre-treatment morphometric features may provide predictors of treatment response approaching the level needed for clinical application, consistent with findings of the two prior smaller studies (Fleck et al., 2017; Wade et al., 2016). We also assessed the ability of acute changes in morphometric features one week following treatment initiation to predict clinical outcome at week 6. The rationale for this aspect of our design is that early drug-induced changes in brain might provide an indication of clinically-relevant effects of drugs before clinical changes themselves can predict outcome of a drug trial. Changes in morphometric features from baseline achieved comparable prediction accuracy for the two study drugs (76.5% for quetiapine group and 78.9% for lithium group), though models were different for the two drugs. Previous studies using somewhat longer re-test periods (2–4 weeks) after drug treatment initiation have shown neuroanatomic changes with short-term acute treatment with lithium (Anand et al., 2020) and antipsychotic drugs (Keshavan et al., 1994). Our findings suggest that relevant changes can be detected even earlier after treatment initiation. A study of lithium treatment in BD reported effects in the hippocampus/amygdala complex after several years, regions known to be important in emotion processing (Germana et al., 2010). Our study extends these earlier findings by showing that effects occurring as early as one week can predict later treatment response. Thus, the monitoring of early drug effects on brain structure may provide a way of predicting treatment response weeks before treatment outcome can be determined clinically, potentially providing important information to clinicians considering adjustments to treatment plans in individuals who are not early treatment responders.

Combining baseline brain features and acute treatment-induced effects (1-week) in models predicting 6-week treatment response modestly improved the accuracy (83.2% for the quetiapine group and 83.5% for the lithium group). Therefore, combining baseline and follow-up data within a single model may provide a promising direction for maximizing early prediction of treatment outcome in individuals with BD. While these findings require replication in an independent sample to confirm predictive utility of the models developed, they offer a promising preliminary step toward the development of clinically useful MRI-based biomarkers for guiding optimal and flexible treatment planning for individuals in the early course of BD.

Given these differences in prediction performance, we sought to identify which morphometric features provide the greatest contribution to treatment outcome. At baseline, cortical thickness features were better predictors of lithium response, while cortical surface area and subcortical volumes were more prominent as features predicting response to quetiapine. In contrast, when considering morphometric changes at week 1, the opposite pattern was observed with changes in cortical surface area and subcortical volume measures being more prominent in lithium response prediction, and changes in cortical thickness measures being more predictive of quetiapine response. According to the radial unit hypothesis and the supra-granular layer expansion hypothesis, cortical thickness is determined by the number and size of cells within a cortical column and surrounding neuropil, while surface area is less dynamic being primarily driven by the number of cortical columns established during brain maturation (Rakic, 2009). This suggests that dynamic illness-related pathophysiological changes may be more related to lithium outcomes, while relatively enduring features of brain development at the baseline may be better predictors of quetiapine response and dynamic changes in cells and neuropil at one week may be more related to quetiapine treatment. Changes in surface area at one week following lithium treatment are less readily interpretable, but may indicate a more global shift in the neocortical mantle following administration of lithium salts (Hozer et al., 2020).

For the regionality of the most discriminative brain morphometric patterns, at baseline, the most discriminative regions for quetiapine were mainly located in temporal and subcortical regions, while frontal and parietal regions predicted treatment outcome better in lithium group. When considering morphometric changes at week 1, the regions contributing most to prediction mainly comprised frontal and temporal regions for both quetiapine and lithium. Previous research had reported disruption in fronto-temporal neural circuitry in remitted patients with BD (Robinson et al., 2009). Our finding may indicate that the acute morphometric changes in fronto-temporal neural circuitry may in part underlie therapeutic treatment effects for both quetiapine and lithium. Nevertheless, we should note the difference in sample size of these two medication groups which may impact precision in the estimation of contribution weight of features. Although our study is designed as a randomized clinical trial to reduce group differences as much as possible, and no significant confounds (i.e. age, sex, IQ, illness duration, YMRS, CDRS-R, etc.) were identified in our analyses, some other potential differences in patient samples to a degree can exist despite randomization. Replication is still needed to determine the extent that differences in patient samples impacted the current findings.

In addition to these general findings, our transferability test for the two treatments was also informative. The results showed that all models failed to reach comparable performance in the transferability test, indicating that the contributing features of the predictive models differed between treatment groups, both for baseline data and for treatment-related change at week 1. While predictive models for the two treatments had similar success, the quite limited transferability of drug-specific models suggests modest level of shared predictors, so that drug class-specific predictive models appear to be needed for treatment outcome prediction. Different changes in morphometric features at week 1 are perhaps not surprising given the markedly different pharmacology of the two study drugs, though the nature of the differences offers insight into regional differences in early drug impact on brain that are relevant to their therapeutic efficacy.

Following recent recommendations on overcoming methodological issues that can lead to inflated results (Arbabshirani, Plis, Sui, & Calhoun, 2017; Wolfers, Buitelaar, Beckmann, Franke, & Marquand, 2015; Woo, Chang, Lindquist, & Wager, 2017), we adopted two methodological precautions. First, to reduce the risk of overfitting and overly optimistic utility prediction estimates (Arbabshirani et al., 2017; Wolfers et al., 2015; Woo et al., 2017) for the high-dimensional neuroimaging data, we first applied DL technology to identify compact hierarchical features and achieve dimension reduction. Second, we examined region-level features with less noise and lower risk of overfitting than voxel-level data (Vieira et al., 2017). Other approaches for dimension reduction and development of classification algorithms may confirm and potentially refine models developed in the current study (Claude, Houenou, Duchesnay, & Favre, 2020; Collins & Moons, 2019).

Of note, there are several limitations in our current study. First, the models described in this paper will require replication in an independent dataset before any application in clinical decision making. Second, each patient in our study received either quetiapine or lithium monotherapy. Thus, our study can only predict response of each drug and identify predictors of response in two independent treatment groups. Information about prediction of individual-level preferential response to quetiapine or lithium cannot be provided, which should be interpreted with caution. Third, our study was not powered to examine the relationship between MRI features and outcome separately in different age groups. Fourth, the utility of predictive models is generalizable within drug class and remains to be evaluated. Fifth, additional outstanding questions include the ability of other imaging modalities, such as DTI and resting-state fMRI, to inform and supplement prediction models. In addition, it would be helpful to further evaluate the utility of our models for predicting treatment outcomes in adults and in individuals with a long history of illness and drug therapy.

In conclusion, the present study demonstrates that brain morphometric features may predict treatment response for two widely used medications for the treatment of youth with BD. Brain morphometric features provided similar overall prediction performance for both lithium and quetiapine therapies, though the features used to achieve that prediction varied between the two medications. In addition, our findings indicate that brain morphometric changes occurring after a single week of medication exposure are predictive of treatment outcome. These findings provide insight into brain morphometric features associated with the treatment outcome prediction in bipolar youth treated with quetiapine and lithium, and support the potential use of neuroanatomical scans as biomarkers for the optimization of treatment or personalized medication approach which offers potential for reducing the risk of a failed medication trial detectable only following a full course of treatment.

## Supplementary material

For supplementary material accompanying this paper visit https://doi.org/10.1017/S0033291722000757. click here to view supplementary material
