# Supplementary material for: Brain morphometric features predict medication response in youth with bipolar disorder: a prospective randomized clinical trial
Source: Psychol Med. 2022 Apr 8;53(9):4083–93. doi: 10.1017/S0033291722000757 (PMC10317810; doi:10.1017/S0033291722000757)
Supplement: Supplementary file 1 [file S0033291722000757sup001.docx]

**Online Supplementary Materials**

**Materials and Methods**

# 1. Processing of structural MRI scans

All MRI scans were processed on the same workstation using FreeSurfer image analysis suite v6.0.0 (http://surfer.nmr.mgh.harvard.edu/). FreeSurfer is a well-validated neuroimaging processing protocol that has previously been described in detail (1, 2). Briefly, raw structural images undergo several preprocessing steps encompassing motion correction, skull stripping, Talairach transformation and intensity harmonization. Individual reconstructed maps are subsequently obtained following segmentation, tessellation and deformation, and finally registered to the standard spherical space via inflation to project gyrus and sulcus divisions onto the surface. All reconstructed images were manually inspected and fixed for topological deficits. Based on reconstructed images, the surface area at each vertex was calculated as the average area of surrounding triangles mapped in a prior cortical surface tessellation. The cortical thickness was defined as the shortest distance between the gray-white matter boundary and pial surface at each vertex. The subcortical volume resulted from an alternative volume-based pipeline which extracted average gray matter probability of voxels in labeled subcortical structures. To obtain unbiased estimates of these morphological measures, images are further processed with the FreeSurfer longitudinal stream after cross-sectional processing. Longitudinal stream initializes the same processing steps with common information from an unbiased within-subject template created by robust and inverse consistent registration (3, 4), significantly increasing reliability and statistical power (5).

# 2. Two-stage prediction model

## 2.1 The rationale for two-stage prediction model based on deep learning

Previous studies have suggested that deep learning, a type of machine learning capable of capturing high orders of complexity and abstraction (6), may yield higher classifier accuracy than the current widely adopted traditional machine learning models (7, 8). It has been reported that brain structure is undergoing a variety of alterations in typically developing youth (9, 10). Considering the additional psychiatric condition and medication effects on the patients, the complexity of brain structural patterns, especially in longitudinal occasions of a prospective study, may be far beyond what linear methods (e.g., principal component analysis, sparse learning) can extract (11). Deep neural network can precisely recognize the most differentiable features related to medication response from the complex longitudinal structural patterns in a non-linear way. Compared with other non-linear methods, deep neural network facilitates the extraction of optimal low-dimensional representations for clinicians unequipped with expert feature engineering knowledge, which are intensively required in most models. Though the traditional neural network can solely implement classification, combining neural network and an additional classifier increases the model flexibility and have been reported to outperform the single classification neural network (11). We therefore utilized a two-stage prediction pipeline that includes a deep neural network component for non-linear dimensionality followed by an additional support vector machine (SVM) classifier as described in a previous prospective study (11).

## 2.2 Neural network architecture

The core of our medication response prediction is the implementation of the neural network for non-linear dimensionality reduction (12). The multi-layer neural network generally includes one input layer, several hidden layers and one output layer. Each layer contains a set of artificial neurons which exactly correspond to the feature representations at different level. The feature representations at different levels are associated by fully connections between neurons of two consecutive layers. These connections between neurons indicate the strength and direction from input to output, enabling the level-by-level information flow via weighted combination. Non-linear activation function (i.e., ReLU, Sigmoid) is additionally applied between two consecutive layers to handle the potential linear inseparability. The neural network iteratively updates weights by backpropagation to minimize the loss between the network outputs and true labels. Therefore, deep neural networks can automatically abstract optimal low-dimensional information from raw features via hierarchical architecture with consecutively decreased neurons in each layer (6). Herein, we implemented a five-layer neural network for non-linear dimensionality reduction in each model. For the baseline model or one-week change model, the numbers of units in hidden layers were set to 75, 15 and 3. Given the longitudinally joint model doubled the number of input features, hidden units were set to 150, 30, 6, which were likewise doubled.

## 2.3 Binary preprocessing of raw features

The raw high dimensional features were represented as a matrix $X\in\mathbb{R}^{m\times d}$ where the element $x_{ij}$ denotes the feature $j$ for subject $i$ and the labels were represented as a $m$-dimensional vector $Y\in{\{0,1\}}^{m}$, with 1 indicating responders and 0 indicating non-responders. Given the common application of binary input in unsupervised two-layer greedy networks (12, 13), we applied binary transformation on high-dimensional real-valued features resulting in a binary matrix $X\in{\{0,1\}}^{m\times d}$. The binary threshold of feature $j$ is calculated as $(m_{0}^{j}+m_{1}^{j})/2$ where $m_{0}^{j}$ denotes the median value of feature $j$ in non-responders and $m_{1}^{j}$ denotes the median value of feature $j$ in responders. The values of feature$j$ less than or equal to the threshold are assigned 0, and those larger than the threshold are assigned 1. To ensure the independence between the training and validation set, the binary transformation threshold was estimated from the training set and applied to the validation set in each cross-validation loop.

## 2.4 Model training

A two-step protocol performs the training of the neural network. In the first step, pre-training is implemented via another unsupervised DL model called autoencoder (AE) with a typical encoder and decoder structure. Unlike traditional deep neural networks, the number of neurons in consecutive layers does not always decrease. The encoder’s architecture is similar to a common neural network, which generates low-dimensional representations, while the decoder utilizes the latent information to reconstruct the original input. Without any given labels, AE takes good advantage of the inherent pattern in data by minimizing the reconstruction error in an unsupervised manner. Moreover, shallow AEs can be stacked into a deeper structure called stacked autoencoders (SAE), enabling the extraction of higher-order latent representations (14). In this case, individual AE is greedily trained, and the optimal latent representation of the previous AE is delivered to the next AE to generate more abstract representation (15). Herein, we trained a SAE model with 4 three-layer AEs (1 input layer, 1 hidden layer and 1 output layer) whose architectures conform to the following supervised deep neural network. Individual AE is thought to be converged when meeting one of the following criteria (11): (1) default 100 epochs are reached; (2) the reconstructed loss measured by mean square error (MSE) for the last 10 epochs is continuously less than 0.001. To weaken the impact of random weight initialization, the training process of SAE repeated 10000 times. The median values of these fine-tuned weights are transferred to the subsequent deep neural network as initial weights for the next supervised step.

In the supervised learning part, a five-layer neural network was trained to fine-tune the weights and biases. The softmax function is additionally applied to project the scalar output into the probability of binary classes for supervised learning. The training loss estimated by cross-entropy function is optimized with an adaptive moment estimation (Adam) optimizer (momentum parameters = [0.9,0.999], initial learning rate = 0.001). Once the training completes, the representations in the layer before the output layer were extracted as the optimal features in low-dimensional space. We subsequently fed the resulting low-dimensional features into a binary linear SVM classifier. During the training process at SVM stage, 5-fold nested cross-validation was performed to find the optimal hyperparameter $C$ from $C={\{10}^{-3}, {10}^{-2},{10}^{-1},1,{10}^{1},{10}^{2},{10}^{3},{10}^{4}\}$ via grid search. Once the optimal hyperparameter for each fold was determined, SVM was trained again with the whole training set and evaluated on the validation set.

# 3. Calculation of feature contribution

We extracted all the weight matrices $W=\{W_{1},W_{2}.W_{3}, W_{4}\}$ connecting consecutive layers $L=\{l_{1},l_{2},l_{3},l_{4}, l_{5}\}$ from the fine-tuned network, where $l_{i}$ denotes the *i-*th layer and $W_{i}$ denotes the weight matrix connecting $l_{i}$ and $l_{i+1}$. As Hazlett et al. described (11), the contribution of each node in a given layer $i$ was estimated from the weight matrix $W_{i}$. We accordingly started from $l_{4}$ and work backwards keeping nodes with greatest contributions whose summed contributions represent more than 50% of the weight contribution in $l_{4}$. Next, node contributions in $l_{3}$ were estimated using a partition of weight matrix $W_{3}$ restricted to those surviving nodes in $l_{4}$. This calculation was propagated backward until we reached the $l_{1}$ where contributions of raw features are available.

| **Characteristic** | **Quetiapine (n = 71)** | **Lithium (n = 50)** |
| --- | --- | --- |
| Lifetime comorbidity, N (%) |  |  |
| Psychosis | 7 (10.9%) | 6 (12.0%) |
| Anxiety disorder | 14 (19.7%) | 7 (14.0%) |
| ADHD | 26 (36.6%) | 14 (28.0%) |
| Lifetime substance abuse, N (%) | 3 (4.2%) | 2 (4.0%) |
| Family history of bipolar diorder^a^, N (%) | 20 (40.8%) | 11 (32.4%) |
| Lifetime medication exposure^a^, N (%) |  |  |
| Antidepressant | 20 (40.8%) | 11 (32.4%) |
| Antipsychotics | 21 (42.9%) | 12 (35.3%) |
| Mood stabilizer | 1 (2.0%) | 0 (0.0%) |
| Psychostimulants | 19 (38.8%) | 13 (38.2%) |
| Benzodiazepines | 0 (0.0%) | 2 (5.9%) |

**Table S1** Supplemental clinical information of included participants.

Abbreviations: ADHD, attention-deficit/hyperactivity disorder

^a^Data were available in 83 of 121 participants

**Supplemental References**

1. Dale AM, Fischl B, Sereno MI. Cortical surface-based analysis. I. Segmentation and surface reconstruction. Neuroimage 1999;9(2):179-194. doi: 10.1006/nimg.1998.0395

2. Fischl B, Sereno MI, Dale AM. Cortical surface-based analysis. II: Inflation, flattening, and a surface-based coordinate system. Neuroimage 1999;9(2):195-207. doi: 10.1006/nimg.1998.0396

3. Reuter M, Fischl B. Avoiding asymmetry-induced bias in longitudinal image processing. Neuroimage 2011;57(1):19-21. doi: 10.1016/j.neuroimage.2011.02.076

4. Reuter M, Rosas HD, Fischl B. Highly accurate inverse consistent registration: a robust approach. Neuroimage 2010;53(4):1181-1196. doi: 10.1016/j.neuroimage.2010.07.020

5. Reuter M, Schmansky NJ, Rosas HD, Fischl B. Within-subject template estimation for unbiased longitudinal image analysis. Neuroimage 2012;61(4):1402-1418. doi: 10.1016/j.neuroimage.2012.02.084

6. LeCun Y, Bengio Y, Hinton G. Deep learning. Nature 2015;521(7553):436-444. doi: 10.1038/nature14539

7. Pinaya WH, Gadelha A, Doyle OM, et al. Using deep belief network modelling to characterize differences in brain morphometry in schizophrenia. Sci Rep 2016;6:38897. doi: 10.1038/srep38897

8. Vieira S, Pinaya WH, Mechelli A. Using deep learning to investigate the neuroimaging correlates of psychiatric and neurological disorders: Methods and applications. Neurosci Biobehav Rev 2017;74(Pt A):58-75. doi: 10.1016/j.neubiorev.2017.01.002

9. Giedd JN, Blumenthal J, Jeffries NO, et al. Brain development during childhood and adolescence: a longitudinal MRI study. Nat Neurosci 1999;2(10):861-863. doi: 10.1038/13158

10. Tamnes CK, Herting MM, Goddings AL, et al. Development of the Cerebral Cortex across Adolescence: A Multisample Study of Inter-Related Longitudinal Changes in Cortical Volume, Surface Area, and Thickness. J Neurosci 2017;37(12):3402-3412. doi: 10.1523/JNEUROSCI.3302-16.2017

11. Hazlett HC, Gu H, Munsell BC, et al. Early brain development in infants at high risk for autism spectrum disorder. Nature 2017;542(7641):348-351. doi: 10.1038/nature21369

12. Hinton GE, Salakhutdinov RR. Reducing the dimensionality of data with neural networks. Science 2006;313(5786):504-507. doi: 10.1126/science.1127647

13. Lee H, Grosse R, Ranganath R, Ng AY. Convolutional deep belief networks for scalable unsupervised learning of hierarchical representations. Proceedings of the 26th Annual International Conference on Machine Learning, ICML 2009, Montreal, Quebec, Canada, June 14-18, 20092009.

14. Vincent P, Larochelle H, Lajoie I, Bengio Y, Manzagol PA. Stacked Denoising Autoencoders: Learning Useful Representations in a Deep Network with a Local Denoising Criterion. Journal of Machine Learning Research 2010;11:3371-3408.

15. Larochelle H, Erhan D, Courville AC, Bergstra J, Bengio Y. An empirical evaluation of deep architectures on problems with many factors of variation. International Conference on Machine Learning2007.
